# Curcumin protects rat hippocampal neurons against pseudorabies virus by regulating the BDNF/TrkB pathway

**DOI:** 10.1038/s41598-020-78903-0

**Published:** 2020-12-17

**Authors:** Bingjie Yang, Guodong Luo, Chen Zhang, Luqiu Feng, Xianmei Luo, Ling Gan

**Affiliations:** 1 College of Veterinary Medicine, Southwest University, Chongqing, 402460 China; 2 Immunology Research Center, Medical Research Institute, Southwest University, Chongqing, 402460 China

**Keywords:** Neuroscience, Neurology

## Abstract

Pseudorabies virus (PRV) infection can elicit nervous system disorders. Curcumin has been reported to have neuroprotective effects. However, whether curcumin can protect neurons against PRV infection and the underlying mechanisms remain unclear. In the present study, for the first time, the protective effects of curcumin against PRV-induced oxidative stress, apoptosis, and mitochondrial dysfunction in rat hippocampal neurons and the brain-derived neurotrophic factor (BDNF)/tropomyosin-related kinase B (TrkB) pathway were investigated. Results indicated that PRV with a titer of 3.06 × 10^6^ TCID50 (50% tissue culture infective dose) induced oxidative damage of hippocampal neurons 2 h post-infection and that 10 μM curcumin improved the viability of PRV-infected hippocampal neurons. Blocking the BDNF/TrkB pathway reversed the neuroprotective effects of curcumin, which were imparted by decreasing the PRV-induced upregulation of nitric oxide synthase expression, repressing the PRV-activated mitochondrial apoptotic pathway, and mitochondrial dysfunction. To conclude, curcumin exhibited a neuroprotective role against PRV infection by upregulating the BDNF/TrkB pathway. This study provides insight into the anti-PRV neuroprotective application of curcumin and the underlying mechanism in the prophylaxis and treatment of neurological disorders caused by PRV infection.

## Introduction

Pseudorabies (PR) is a highly contagious viral disease caused by the pseudorabies virus (PRV), which results in considerable economic losses to the pig industry^1^. PRV is an enveloped double-stranded DNA virus belonging to the Alphaherpesvirus sub-family in the Herpesviridae family, and is the etiological agent of Aujeszky’s disease in pigs^2^. With the exception of humans and higher primates, the virus can infect almost all mammals, even though the pig is its natural host. PRV infection is characterized by respiratory, reproductive, and neurological symptoms, which depend on the age of the pig and the virulence of the strain. Importantly, PRV infection dramatically causes nervous system disorders and high mortality in newborn piglets^3^. It is worth mentioning that PRV has been accepted as a model system to investigate herpesviruses in neurophysiology and pathogenesis^4^.

A previous study showed that PRV infection affected mitochondrial function^3^. Mitochondria are essential for providing cellular energy (ATP), regulating oxidative stress, apoptosis^5^, and producing reactive nitrogen species (RNS), including nitric oxide (NO)^6^. Increased nitroxidative stress promotes mitochondrial dysfunction^7^, which is implicated in various neurodegenerative diseases and neuropathies^8^. Viral infection has been associated with oxidative stress by decreasing antioxidant enzyme activity and increasing the release of reactive oxygen species (ROS) and/or RNS. Furthermore, PRV causes mitochondrial-dependent apoptosis in cultured cells and neural tissue of swine^9^. This suggests that oxidative stress, mitochondrial dysfunction, and neuronal apoptosis are the key factors implicated in neurodegenerative diseases and neuropathies related to PRV infection, and these are the targets of neuroprotective agents aimed to control PRV infections.

Curcumin, the primary component of *Curcuma longa*, is an acidic phenolic substance which has been reported to have many pharmacological activities, including anti-inflammatory, antioxidant, anti-proliferative, and neuroprotective effects^10^. Moreover, it has been reported to have anti-viral activity against dengue (serotype 2)^11^, herpes simplex virus^12^, and vesicular stomatitis virus^13^. However, the use of curcumin to protect neurons against PRV infection has rarely been studied^14^. Curcumin seems to be a promising agent for treating neurodegenerative diseases due to its ability to cross the blood–brain barrier^15^. Consequently, we hypothesized that curcumin may alleviate the neuronal damage induced by PRV infection, and that mitochondria-related oxidative stress and the apoptotic pathway may be the underlying factors implicated in the process^8^.

Brain-derived neurotrophic factor (BDNF) is a well-known neurotrophic factor with critical functions in neuronal plasticity, regulation of mitochondrial transport and distribution, and neuronal protection, including anti-apoptosis, anti-oxidation, and suppression of autophagy^16^. BDNF plays important regulatory roles primarily by binding to transmembrane tropomyosin-related kinase B (TrkB) localized to mitochondrial membranes^17^. Stimulant-induced brain damage has been shown to reduce BDNF protein levels in the brain^18^, while increased BDNF levels resisted mitochondrial dysfunction induced by 3-nitropropionic acid in primary rat cortical cultures^16^. Thus, PRV infection can also lead to changes in BDNF levels in hippocampal neurons. Since the BDNF/TrkB pathway has been reported to prevent neuronal cell death induced by various stimulants and impart neuroprotection^19,20^, we hypothesized that the BDNF signaling pathway may mediate the neuroprotective effects of curcumin against PRV infection.

Therefore, this study was designed to investigate the neuroprotective role of curcumin against PRV infection and elucidate its underlying mechanisms. To the best of our knowledge, this is the first study to report the neuroprotective effects of curcumin against PRV-induced oxidative stress, apoptosis, and mitochondrial dysfunction in rat hippocampal neurons and demonstrates that the underlying mechanism involves the upregulation of BDNF/TrkB signaling pathway.

## Materials and methods

### Isolation and culture of rat hippocampal neurons

All procedures were approved by the Animal Care Committee of Southwest University and were performed in compliance with the National Institutes of Health guide for the care and use of Laboratory animals (NIH Publications No. 8023, revised 1978). Primary hippocampal neurons were isolated as described previously^21^ with some modifications. Hippocampi isolated from the brains of neonatal Sprague Dawley rats (< 24 h) were sliced using ophthalmic scissors under sterile conditions. The tissues were incubated in calcium- and magnesium-free Hanks’ balanced salt solution at 37 °C for 2 min and then re-suspended in Dulbecco’s modified eagle medium (DMEM) (Invitrogen, Carlsbad, CA, USA) supplemented with 10% fetal bovine serum (FBS, Gibco BRL, Grand Island, NY, USA). Neurons were plated at the density of 4–5 × 10^5^ cells/mL on poly-d-lysine pre-coated plastic dishes. Cultures were maintained in serum-free B27/Neurobasal medium (Gibco BRL, Grand Island, NY, USA) in an incubator (95% air, 5% CO_2_) at 37 °C. On the third day, 2 μg/mL cytosine β-d-arabinofuranoside (AraC, Sigma-Aldrich, St. Louis, MO, USA) was added to inhibit the non-neuronal cell proliferation. On day 5 in vitro, more than 95% of the cells had differentiated into neurons. Hippocampal neurons were chosen for experiments between days 7–9 of in vitro culture, and the purity was assessed by immunocytology to ensure a gliocyte percentage < 5%.

### Virus infection and titration

Chongqing Academy of Animal Science provided the porcine kidney cell line PK-15. The cells were maintained in DMEM containing 10% FBS (Gibco) and antibiotics (0.1 mg/mL streptomycin and 100 IU/mL penicillin). For the maintenance medium (MM), the serum concentration was reduced to 2%. The cells were incubated at 37 °C in an incubator with a humidified atmosphere of 5% CO_2_. PRV (Rong A strain, China Veterinary Culture Collection Center) was propagated in PK-15 cells cultured in MM and then stored at − 80 ℃. PK-15 cells were grown to 90% confluency and were infected with PRV at various multiplicities of infections. The mock-infected cells were treated with MM. After 1 h, the inoculum was removed by aspiration. Cells were then washed twice with PBS to remove any virus particles that did not cause infection. The cells were incubated in MM at 37 °C for various times until harvesting. The diluted virus was subsequently titered in PK-15 cells to confirm the final titer. The TCID50 was calculated using the Reed–Muench method^22^.

### Measurement of nitric oxide level, malondialdehyde level, and superoxide dismutase activity

Hippocampal neurons were seeded in 6-well plates at the density of 4 × 10^5^ cells/mL for 2 mL culture medium. After treatment with a virus titer of 3.06 × 10^6^ TCID50 PRV for different times, the cells were harvested to estimate the NO level, malondialdehyde (MDA) level, and superoxide dismutase (SOD) activity using the corresponding assay kits (Nanjing Jiancheng Bio Company, China).

### Assay for cell viability and lactate dehydrogenase (LDH) leakage

Primary hippocampal neurons were seeded into 96-well plates at a density of 10,000 cells/well and incubated in 200 μL neurobasal medium with 2% B-27 (Thermo Fisher Scientific, Italy) supplement for 7 days. Curcumin with a purity of 98% (GR0580; Nanjing Guangrun Biotechnology) was prepared as a 1 mmol/mL stock solution in dimethyl sulfoxide (DMSO) and stored at − 20 °C away from light. DMSO with a concentration lower than 0.5% (v/v) had no effect on the neurons or PRV proliferation^21^. Throughout the test, the final concentration of DMSO was 0.1% (v/v). For anti-viral assays, curcumin was diluted to different concentrations in neurobasal medium. After dilution, hippocampal neurons were treated with 2.5-, 5-, 10-, 20-, 40-, and 60-µM curcumin for 24 h. Thereafter, cell counting kit-8 (CCK-8) solution was added into each well, and the absorbance of each well was recorded at 450 nm using a microplate reader. In the LDH assay, neurons were pretreated with 0-, 2.5-, 5-, 10-, 20-μM curcumin for 24 h and incubated with a virus titer of 3.06 × 10^6^ TCID50 PRV for 2 h. Neuronal injury was subsequently assessed by estimating the released LDH using an LDH assay kit (Nanjing Jiancheng Biotechnology Institute, China).

### JC-1 assay for mitochondrial membrane potential

Mitochondrial membrane potential (MMP) in hippocampal neurons was measured using an MMP assay kit with a cationic dye, JC-1, that accumulates in functional mitochondria (Beyotime Biotechnology, Shanghai, China). When the mitochondrial membrane potential is high, JC-1 accumulates in the matrix of mitochondria to form a polymer, which produces red fluorescence, whereas, when the mitochondrial membrane potential is low, JC-1 cannot gather in the matrix of mitochondria; at this time, JC-1 is a monomer and produces green fluorescence. Therefore, the change in mitochondrial membrane potential can be detected by the change in fluorescence color. The relative ratio of red to green fluorescence is often used to measure the ratio of Mitochondrial depolarization.

After being seeded into 6-well plates, hippocampal neurons were incubated with PRV for 2 h with or without 10 μM curcumin pretreatment for 24 h and 10 nM ANA-12 (S7745; TrkB selective antagonist; Selleckchem, Houston, TX, USA) for 20 min before PRV infection. Thereafter, the neurons were collected for fluorescence spectrophotometry analysis and fluorescence microscope observation. The fluorescence intensities were measured using a fluorescence plate reader at 590 nm (red) and 529 nm (green). According to the ratio of fluorescence intensities at 590 and 529 nm, the loss of MMP was assessed and recorded.

### Western blotting assay

Caspase-3 activity was detected using a caspase-3 activity assay kit (Beyotime Institute of Biotechnology, Jiangsu, China), and the expression levels of BDNF, TrkB, neuronal nitric oxide synthase (nNOS)*,* and apoptosis-related proteins were investigated by Western blotting^23^. Briefly, hippocampal neurons were harvested after treatment and lysed with RIPA lysis buffer (0.1% SDS, 1% sodium deoxycholate, 1% Triton X-100, and sodium orthovanadate; Beyotime Institute of Biotechnology, Shanghai, China) on ice for 30 min. The lysates were centrifuged at 12,000 rpm for 10 min at 4 °C. Total protein concentration was measured using a BCA protein assay kit (Nanjing Jiancheng Bio-Technology Co., Ltd.). The protein samples were separated on 10% sodium dodecyl sulfate–polyacrylamide gels (SDS-PAGE) and transferred onto a pre-active polyvinylidene fluoride membrane (Merck KGaA, Darmstadt, Germany). Membranes were blocked with a tris buffered saline tween (TBST) buffer containing 5% fat free milk for 2 h and then incubated with specific primary antibodies overnight at 4 °C. After washing three times with TBST buffer, each membrane was incubated at room temperature for 2 h with horseradish peroxidase-conjugated secondary antibodies, and then washed three times with TBST buffer. An enhanced chemiluminescence kit (ECL; Beyotime, China) was used to visualize immunoblots. The immunoreactive bands were analyzed by the Image J system software. β-actin was used as a loading control. Antibodies used in western blotting experiments were as follows: nNOS (1:1000; Cell Signaling Technology, Beverly, MA, USA), TrkB (1:500; Proteintech, Rosemont, IL, USA), BDNF (1:500; Proteintech, Rosemont, IL, USA), Bax (1:2000; Proteintech, Rosemont, IL, USA), Bcl-2 (1:1000; Proteintech, Rosemont, IL, USA), and β-actin (1:2000; Proteintech, Rosemont, IL, USA). The membranes were incubated for 1 h with secondary horseradish peroxidase-conjugated anti-rabbit IgG antibody (1:5000; Proteintech, Rosemont, IL, USA).

### Determination of hippocampal neuron ATP levels

Thereafter, ATP levels were measured using a firefly luciferase-based ATP assay kit (Beyotime Institute of Biotechnology, Jiangsu, China). After rinsing with PBS, the neurons were schizolysized by ATP detection lysis solution and then centrifuged at 12,000×*g* at 4 °C for 5 min, and the supernatant was collected. 100 µL of the supernatant was mixed with 100 µL of ATP detection solution in a 1.5 mL tube. Luminance in relative light units (RLU) was immediately measured using a Turner Bio-systems luminometer. Total ATP levels are presented as RLU (nmol/mg).

### Statistical analyses

All data were analyzed using SPSS (IBM SPSS Statistics, Version 22.0, IBM Corp., Armonk, NY, USA). The results are presented as mean ± standard deviation (SD) of three biological replicates (n = 3). Statistical significance was determined using one-way analysis of variance (ANOVA) followed by a least significant difference (LSD) post hoc test for multiple comparisons among the groups and a two-tailed independent t-test was used to compare the two groups. The differences with P < 0.05 were considered statistically significant.

## Results

### Oxidative damage of hippocampal neurons induced by PRV infection

**Figure 1:**
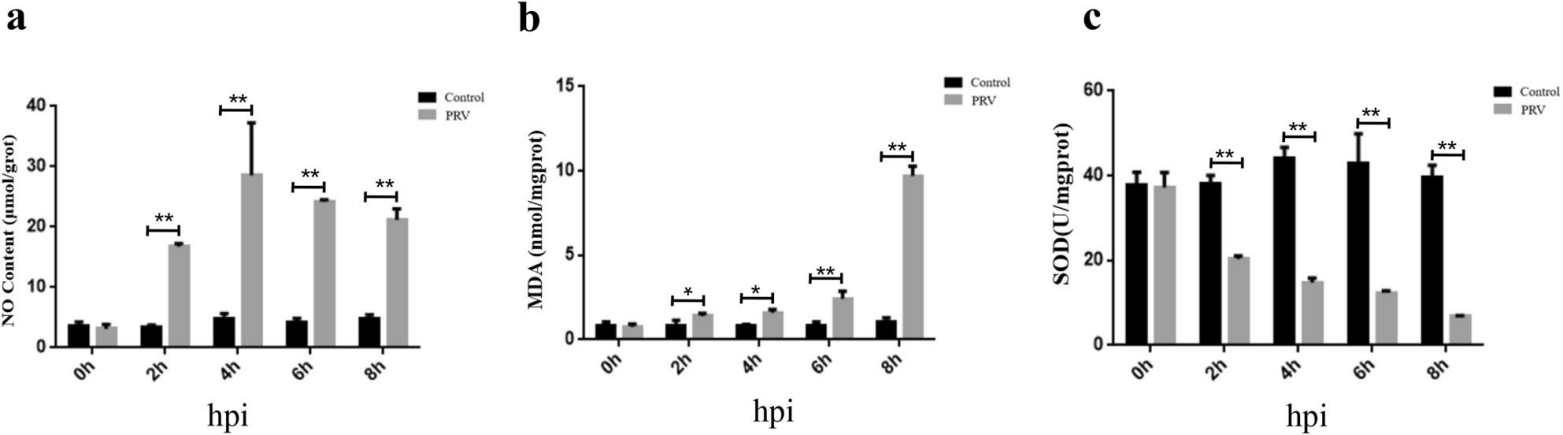
Effects of the duration of pseudorabies virus (PRV) infection on oxidative stress-related markers in hippocampal neurons. (**a**) MDA level; (**b**) NO level; (**c**) SOD activity. *MDA* malondialdehyde, *NO* nitric oxide, *SOD* superoxide dismutase, *hpi* hours post-infection. n = 3, represents three biological replicates, each performed in triplicate. *P < 0.05; **P < 0.01, based on the two-tailed independent-t test.

The MDA level, NO level, and SOD activity were estimated to evaluate the extent of PRV-induced oxidative damage in hippocampal neurons. As shown in Fig. 1a, compared with the control, the NO levels in neurons increased at 2 h, 4 h, 6 h and 8 h after PRV infection (P < 0. 01) (Fig. 1a); additionally, the MDA levels in hippocampal neurons increased at 2 h, 4 h, 6 h and 8 h ( P < 0.05; P < 0.05; P < 0.01; P < 0.01) after PRV infection, while the SOD activity decreased at 2 h, 4 h, 6 h and 8 h after PRV infection (P < 0.01) (Fig. 1b,c). These results suggest that 2 h was the minimum time of infection that caused oxidative damage in hippocampal neurons.

### Cytotoxic and neuroprotective effects of curcumin

**Figure 2:**
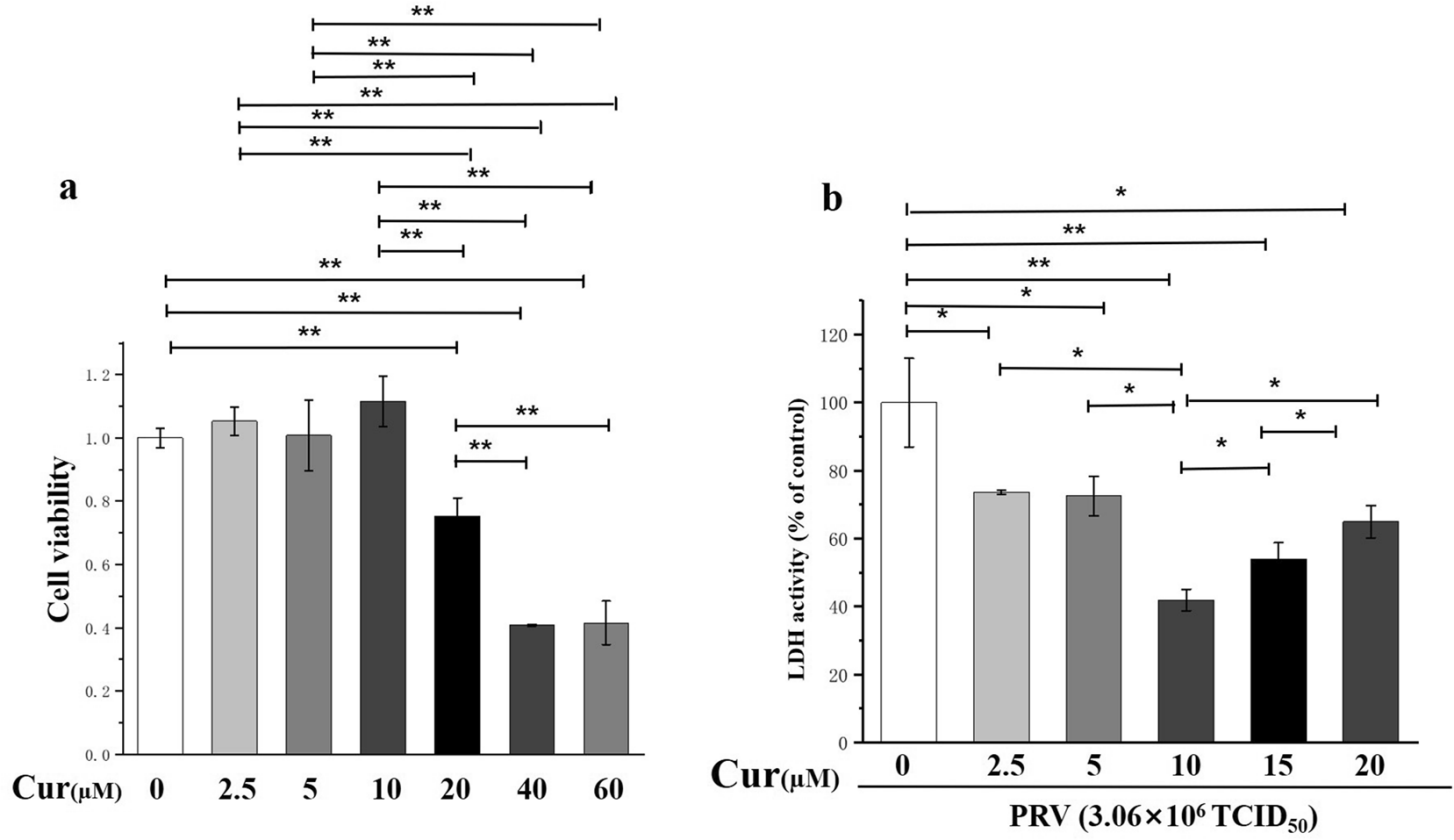
Toxicity and neuroprotective effect of curcumin doses on hippocampal neurons. (**a**) Toxicity of curcumin doses on hippocampal neurons; (**b**) Neuroprotective effect of curcumin doses against hippocampal neurons infected by pseudorabies virus (PRV). n = 3, represents three biological replicates, each performed in triplicate. *P < 0.05; **P < 0.01, based on one-way analysis of variance (ANOVA) followed by least significant difference (LSD) post hoc test for multiple comparisons.

A range of concentrations at which curcumin may exhibit potential cytotoxic activity on hippocampal neurons was tested^24^. Compared to the vehicle control group, the concentrations below 10 µM had no effect on the viability of hippocampal neurons, but doses higher than 20 µM (40 and 60 µM) were toxic to the neurons (Fig. 2a). Therefore, curcumin pretreatment for 24 h with concentrations less than 20 µM and PRV infection with a virus titer of 3.06 × 10^6^ TCID50 for 2 h were used to investigate the neuroprotective role of curcumin against PRV infection. Curcumin pretreatment attenuated the increase of LDH activity (P < 0.05) and had the most noticeable influence at 10 μM, which was 58.04% LDH activity of that of the control group (P < 0.01), while the doses of 5 μM and 15 μM were less effective than 10 μM in reducing LDH activity (Fig. 2b), thus indicating that 10 μM was the optimal dose of curcumin to protect neurons from injury caused by PRV infection.

### Curcumin inhibited the PRV-induced decrease of the BDNF levels

**Figure 3:**
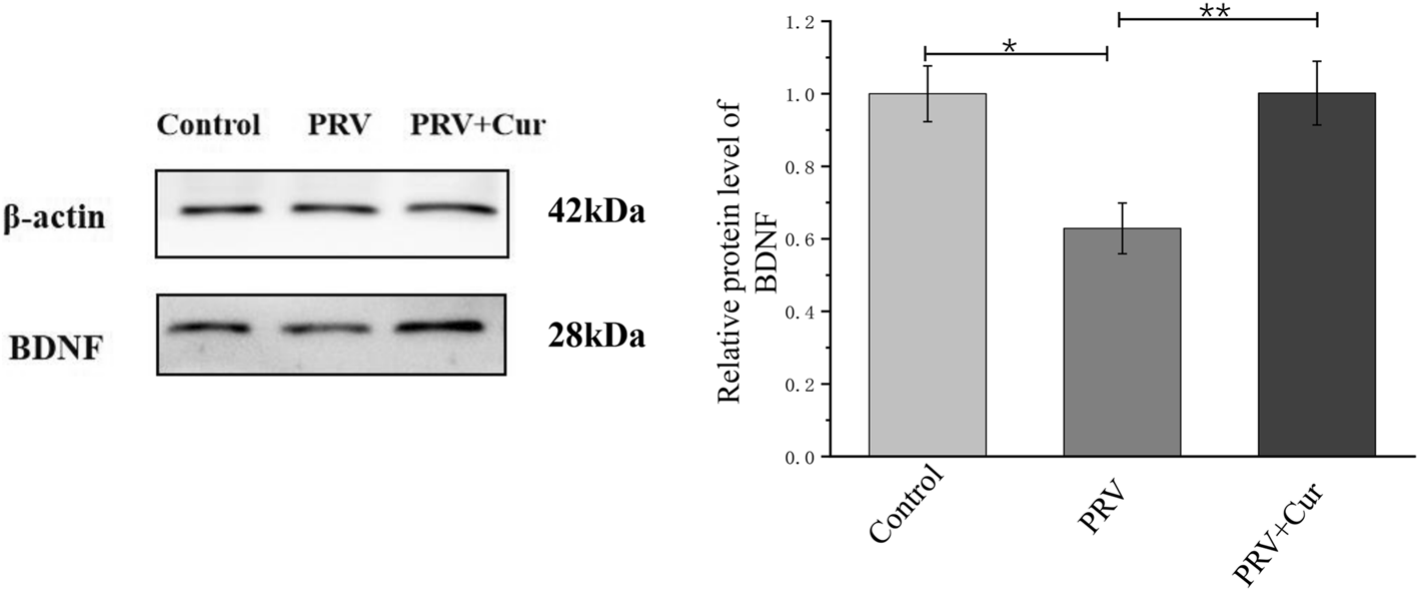
The protein levels of BDNF in hippocampal neurons. The intensity of BDNF bands was normalized with that of β-actin bands detected in the same blot. *Cur* curcumin, *BDNF* brain-derived neurotrophic factor. n = 3, represents three biological replicates, each performed in triplicate. *P < 0.05, **P < 0.01, based on one-way analysis of variance (ANOVA) followed by least significant difference (LSD) post hoc test for multiple comparisons.

BDNF protein levels were measured to determine if curcumin protects hippocampal neurons against PRV infection by regulating the expression of BDNF. Western blotting analysis showed that relative to the control group, PRV-infected neurons had lower BDNF levels (37.12% of control, P < 0.05), while pretreatment with 10 μM curcumin inhibited the decrease in BDNF levels (P < 0.01) (Fig. 3).

### Blocking BDNF/TrkB pathway reversed the neuroprotective effects of curcumin

**Figure 4:**
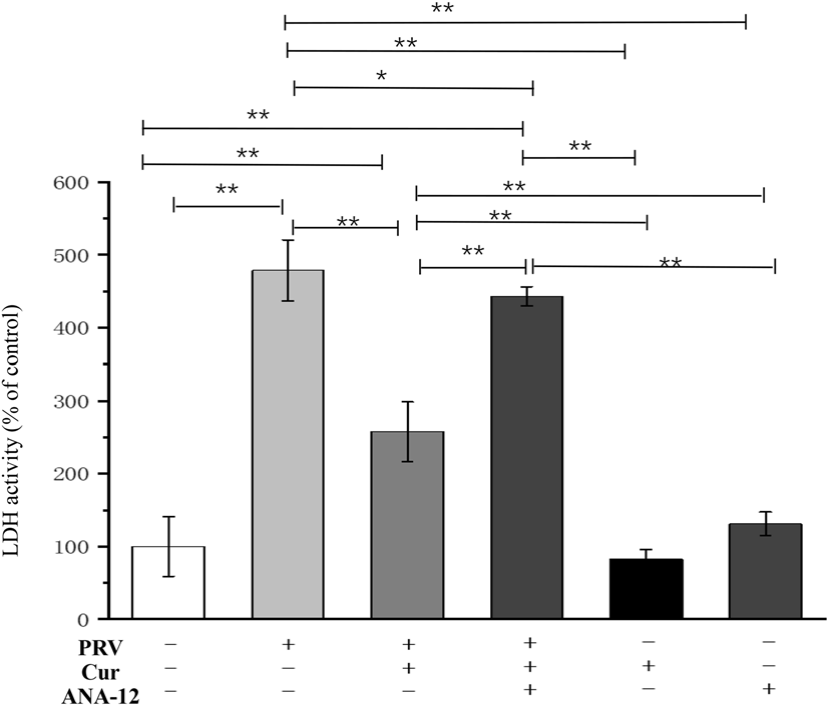
Blocking the BDNF receptor TrkB reversed the neuroprotective effects of curcumin. n = 3, represents three biological replicates, each performed in triplicate. *Cur* curcumin, *BDNF* brain-derived neurotrophic factor, *TrkB* tropomyosin-related kinase B. *P < 0.05; **P < 0.01, based on one-way analysis of variance (ANOVA) followed by least significant difference (LSD) post hoc test for multiple comparisons. “+” means added, “−” means not added.

The TrkB inhibitor, ANA-12, was used to further investigate the involvement of the BDNF/TrkB pathway in the neuroprotective role of curcumin. The LDH assay showed that LDH activity increased by approximately 470% compared to that of the control neurons after exposure to PRV for 2 h. This increase was almost reversed by pretreatment with curcumin for 24 h. The survival-promoting action of 10 μM curcumin was significantly inhibited by the administration of ANA-12 (100 nM) (P < 0.01, vs. curcumin pretreatment group). In contrast, the addition of curcumin or ANA-12 alone did not affect neuronal LDH activity (Fig. 4). The results suggest that the BDNF/TrkB pathway mediates the neuroprotective role of curcumin against PRV infection.

### Curcumin decreased the PRV-induced upregulation of nNOS expression and repressed the PRV-activated mitochondrial apoptotic pathway through the BDNF-TrkB pathway

**Figure 5:**
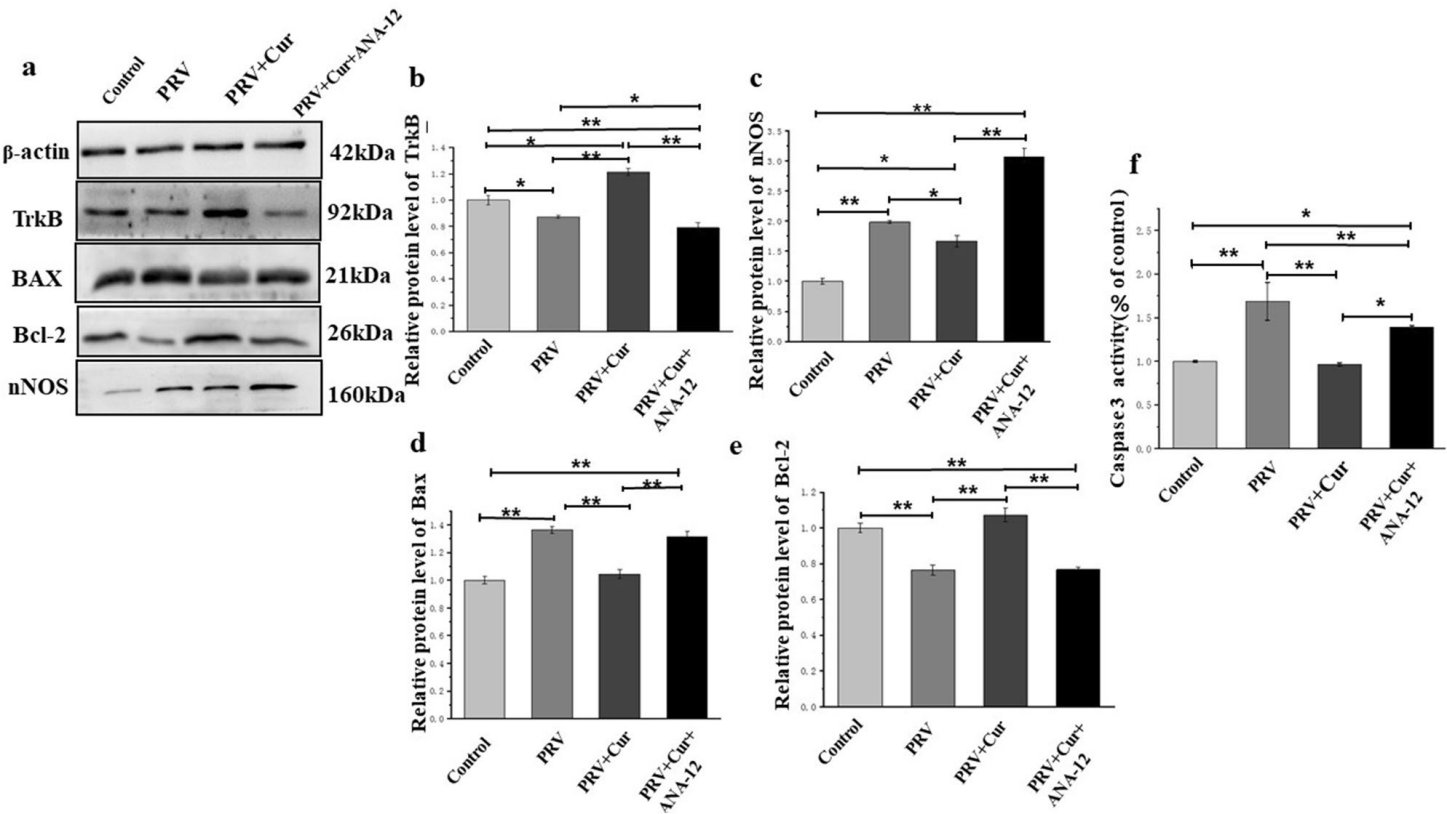
The protein levels of nNOS, TrkB, Bcl-2, Bax, and caspase-3 activation in hippocampal neurons. (**a**) nNOS, TrkB, Bcl-2, and Bax protein levels in hippocampus neurons were measured by western blotting, then intensity was calculated using β-actin as an internal control to normalize protein loading (**b**–**e**). (**f**) caspase-3 activation. *nNOS* neuronal nitric oxide synthase, *TrkB* tropomyosin-related kinase B, *Bcl-2* B-cell lymphoma-2, *Bax* Bcl-2-associated X. n = 3, represents three biological replicates, each performed in triplicate. *P < 0.05, **P < 0.01, based on one-way analysis of variance (ANOVA) followed by least significant difference (LSD) post hoc test for multiple comparisons.

TrkB expression was first detected to validate the inhibitory effect of ANA-12 and to confirm its role as a mediator in the neuroprotective effects of curcumin. The present results indicate that PRV infection reduced TrkB protein levels (P < 0.05), while curcumin pretreatment mitigated the reduced expression of TrkB induced by PRV (P < 0.01). It is noteworthy that the TrkB protein level in PRV + Curcumin group was higher than the control. After ANA-12 administration, the effect of curcumin was blocked (P < 0.01) (Fig. 5a,b), indicating that ANA-12 inhibited TrkB, and that curcumin plays a neuroprotective role by preventing the decrease in TrkB levels induced by PRV. Compared with the vehicle-treated control neurons, neurons infected with PRV showed an upregulation of nNOS (P < 0.01). Pretreatment with curcumin inhibited PRV-induced upregulation of nNOS, suggesting that curcumin reduces PRV-induced NO by blocking the expression of nNOS. However, after pretreatment with ANA-12 (100 nM for 20 min), the expression of nNOS was increased (P < 0.05) (Fig. 5a,c).

Relative to the neurons in the control group, neurons in the PRV infection group had higher Bax levels (Fig. 5a,d), lower Bcl-2 protein levels (Fig. 5e), and higher Caspase-3 activity (Fig. 5a,f) (P < 0.01). In the curcumin pretreatment with PRV infection group, the Bax levels and Caspase-3 activity were lower, and the Bcl-2 levels were higher than those in the PRV infection group without curcumin treatment (P < 0.01). Relative to the neurons in the PRV infection with curcumin pretreatment group, the Bax levels and Caspase-3 activity were higher, and the Bcl-2 levels were lower in the curcumin + PRV + ANA-12 treatment group (P < 0.01). Cumulatively, these results indicate that curcumin decreases the PRV-induced upregulation of nNOS and apoptosis-related protein, Bax, and increases the PRV-induced downregulation of anti-apoptosis-related protein, Bcl-2. Furthermore, these effects were reversed by blocking the BDNF/TrkB pathway.

### Curcumin inhibited PRV-induced mitochondrial dysfunction, and the inhibition of BDNF-TrkB pathway reversed this effect

**Figure 6:**
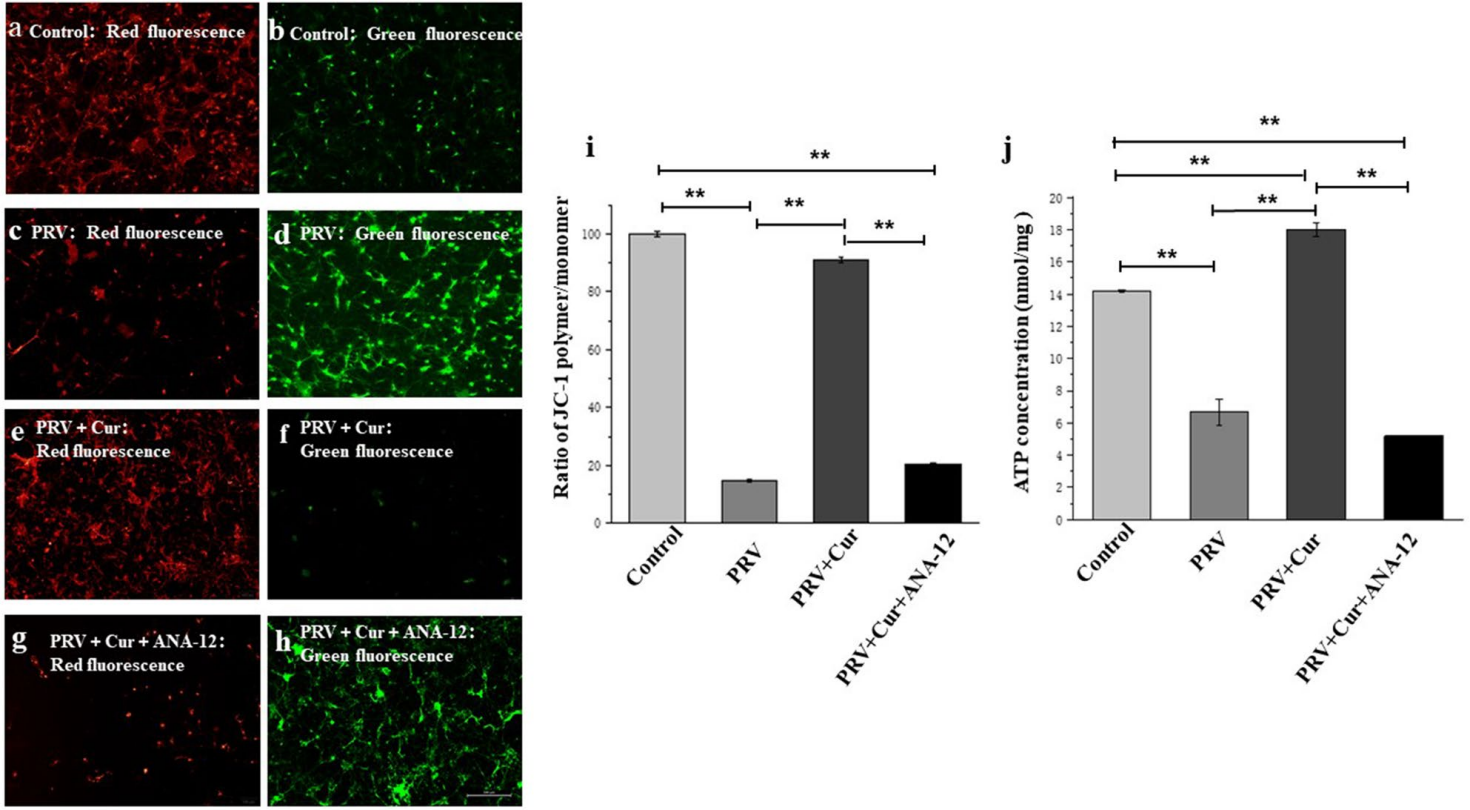
TrkB inhibitor, ANA-12 inhibited the protective effect of curcumin against mitochondrial dysfunction induced by pseudorabies virus (PRV). Red fluorescence represents the mitochondrial aggregate form of JC-1, indicating intact mitochondrial membrane potential. Green fluorescence represents the monomeric form of JC-1, indicating dissipation of mitochondrial membrane potential. (**a**,**b**) Control group; (**c**,**d**) PRV infection group; (**e**,**f**) PRV + Cur group; (**g**,**h**) PRV + Cur + ANA-12 group. (**i**) Quantitative analysis of the shift of mitochondrial orange-red fluorescence to green fluorescence among all groups. The individual red and green average fluorescence intensities are expressed as the ratio of red to green fluorescence. An increase in the bar indicates a shift in the fluorescence ratio correlating with an increase in mitochondrial depolarization. (**j**) ATP concentrations of hippocampal neurons. *Cur* curcumin. n = 3, represents three biological replicates, each performed in triplicate. *P < 0.05; **P < 0.01, based on one-way analysis of variance (ANOVA) followed by least significant difference (LSD) post hoc test for multiple comparisons.

The MMP (Δψm) and ATP levels in hippocampal neurons were measured to elucidate the molecular mechanism underlying the neuroprotective role of curcumin. After 2 h of PRV infection, the Δψm of hippocampal neurons (Fig. 6a,b,i) was lower than curcumin pretreated (Fig. 6c,d,i) and untreated neurons (Fig. 6e,f,i; P < 0.01). However, the Δψm decreased after treatment with ANA-12 (100 nM for 20 min; Fig. 6g,h,i) compared to the curcumin pretreated neurons. Neuronal ATP content is also a sensitive indicator of mitochondrial function. After 2 h of PRV infection, a drastic decrease in cellular ATP levels from 14.17 ± 0.87 nmol/mg protein to 6.67 ± 0.81 nmol/mg protein was observed. In contrast, the treatment of cultures with curcumin resulted in neuronal ATP levels of 18.01 ± 0.44 nmol/mg protein (P < 0.01), which was higher than that of controls. These findings demonstrate that curcumin protects mitochondrial function during PRV infection. However, after treatment with ANA-12 (100 nM for 20 min), the level of ATP in neurons decreased sharply to 5.19 ± 0.32 nmol/mg protein (P < 0.01, Fig. 6j), indicating that the BDNF/TrkB pathway mediates the neuroprotective role of curcumin against the mitochondrial dysfunction induced by PRV.

## Discussion

Regardless of the global vaccination programs, PRV outbreaks are still reported in domestic pigs and wild boars in many countries^25^. Thus, the identification of powerful and safe therapeutic agents is still needed. In the present study, the neuroprotective role of curcumin against PRV infection was evaluated in hippocampal neurons of Sprague–Dawley rats. The present findings demonstrated that 10 μM curcumin pretreatment did not cause neuronal toxicity. Instead, curcumin pretreatment remarkably increased the neuronal viability and reduced LDH release as compared to the neurons infected with PRV. Furthermore, curcumin pretreatment inhibited the PRV-induced upregulation of nNOS, neuronal apoptosis, and mitochondrial dysfunction. Notably, curcumin attenuated the downregulation of BDNF and TrkB induced by PRV. Moreover, blocking of the BDNF/TrkB pathway by the TrkB inhibitor, ANA-12, noticeably reversed the neuroprotective role of curcumin. Collectively, these findings demonstrate that curcumin exhibits neuroprotective effects against PRV infection through the BDNF/TrkB pathway.

PRV is a neurotropic swine Alphaherpesvirus and is the causative agent of various neurological disorders and diseases^26^. Previous research has shown that viral infection causes oxidative stress by triggering the release of excessive ROS/RNS and/or less antioxidant activity^27^. Respiratory syncytial virus infection in BEAS-2B cells has been reported to significantly increase the NO and MDA content^27^. The present study demonstrated similar results, wherein, the infection with PRV for only 2 h induced oxidative damage of hippocampal neurons as evident from increased NO and MDA content, and decreased SOD activity. In the case of PRV infections, an increase in the number of neurons expressing nNOS in the virus-infected area has been reported^28^. As a possible underlying mechanism for PRV-induced neurological disorders, nNOS has been implicated in DNA damage in a mouse model of Parkinson’s disease^29^. These cellular responses have been attributed to excessive NO production by nNOS under neuroinflammatory conditions caused by lipid peroxidation, leading to MDA formation and neuronal death^29^. Thus, it is not surprising to observe the increase in nNOS, NO, and MDA levels and the decrease in neuronal viability caused by the PRV infection in the present study. The PRV infection model system provided us with an opportunity to explore the neuroprotective effect of curcumin, which may be related to its antioxidant activity.

Curcumin is ubiquitous in plants, and its regular consumption has wide medicinal applications^30^. Previous studies have identified that curcumin prevented cellular oxidative stress by acting as an upstream therapeutic barrier to oxidative stress and apoptosis, thereby offering a novel approach to prevent oxidative stress-induced pathogenesis^30,31^. It is well documented that curcumin scavenges the virus-induced oxidative stress in different experimental models^32^. Similarly, in the present study, 10 μM curcumin pretreatment for 24 h inhibited the PRV-induced upregulation of nNOS and subsequent reduction of neuronal viability, indicating that curcumin can protect hippocampal neurons from PRV-induced oxidative stress. Furthermore, we also observed that 5 μM and 15 μM concentrations were less effective than 10 μM curcumin in protecting neurons from injury, which may be due to the fact that 5 μM concentration was too less to protect neurons against injury, and 15 μM concentration of curcumin caused some degree of cytotoxicity.

Dysfunctional mitochondria are less efficient ATP producers but more efficient ROS producers^33^. Furthermore, mitochondrial membrane integrity is essential for normal cell function, and the disruption of mitochondrial function has been implicated in a variety of neurodegenerative diseases and neuropathies^8^. Many viruses and bacteria directly or indirectly destroy the mitochondrial membranes’ integrity and function^5^. In the present study, curcumin pretreatment helped in preventing PRV-induced mitochondrial dysfunction, which was supported by the increase in ATP level and MMP, another important marker for mitochondrial dysfunction. Upon exposure of cells to oxidative stress, the integrity of the mitochondrial membrane is destroyed and mitochondrial function is disrupted, which induces a cascade of signaling events leading to apoptosis^33^. Therefore, mitochondrial dysfunction is an excellent indicator of apoptosis^7^. Classically, caspase-3 has been regarded as the major executor caspase that causes apoptosis^34^. Bcl-2 can negatively regulate cell death and protect cells from sustained exogenous stimuli-induced apoptosis^35^. Conversely, as a pro-apoptotic regulatory protein, Bax can enhance cell death^36^. Under pathological conditions, Bcl-2 weakens apoptosis by impeding the decrease of MMP, and Bax facilitates apoptosis by initiating mitochondrial depolarization^37^. The results of the present study showed that curcumin inhibited Bcl-2 downregulation and Bax upregulation at the protein level, and alleviated the upregulation of caspase-3 activity in PRV-infected hippocampal neurons. This suggests that curcumin can protect hippocampal neurons from PRV-induced mitochondrial dysfunction and apoptosis.

BDNF is deeply involved in the pathophysiology and treatment of mitochondrial dysfunction^38^. As a neuroprotective agent, increased BDNF can rescue neurons from various insults by binding to the TrkB receptor^17^. BDNF plays an important role in counteracting oxidative stress, mitochondrial dysfunction, and apoptosis^16^, thereby suggesting that the suppression of TrkB receptor may contribute to BDNF-mediated neuroprotection. In the present study, we found that curcumin upregulated the expression of BDNF in hippocampal neurons. The BDNF gene has a rather complex structure and can express different mRNA isoforms through alternative splicing, producing 28- to 37-kDa precursors. The mature form is expected to be approximately 14 kDa (monomer) and the dimer is approximately 28 kDa^39–42^. Therefore, the 28-kDa protein in Fig. 3 is likely a dimer of BDNF. Furthermore, blocking the BDNF/TrkB pathway by the inhibition of TrkB reversed the inhibitory effect of curcumin on the upregulation of nNOS, mitochondrial dysfunction, and apoptosis pathway caused by PRV infection. Cumulatively, the present findings provide novel insights into the mechanisms underlying the curcumin-mediated neuronal protection against PRV infection. However, the present study does not fully investigate the different isoforms of BDNF and TrkB, as well as the BDNF/TrkB -mediated signaling pathways activated by curcumin, representing a critical weakness of the study itself. In addition, the results of the present study suggest that the TrkB and ATP levels of PRV infected neurons treated with curcumin were significantly higher than those in the control group, thereby, suggesting that curcumin has strong effects on TrkB and ATP levels independent of PRV-infection conditions, although the exact underlying reason needs further elucidation. Additionally, future studies should further investigate the safety and protective effects of curcumin on the neural tissue of piglets infected with pseudorabies virus in vivo, where the signaling pathways and mechanisms are more complex.

## Conclusion

We demonstrated the neuroprotective effects of curcumin against PRV infection, which were mediated by the regulation of the BDNF/TrkB pathway. These findings suggest that curcumin has potential therapeutic value in the treatment of neurological disorders caused by PRV infection.

## Supplementary Information

Supplementary Information.

## Abbreviations

ATP: Adenosine triphosphate
BDNF: Brain-derived neurotrophic factor
CCK-8: Cell counting kit-8
DMEM: Dulbecco’s modified eagle’s medium
DMSO: Dimethyl sulfoxide
FBS: Fetal bovine serum
LDH: Lactate dehydrogenase
MDA: Malondialdehyde
MM: Maintenance medium
MMP: Mitochondrial membrane potential
nNOS: Neuronal nitric oxide synthase
NO: Nitric oxide
PBS: Phosphate buffer saline
PRV: Pseudorabies virus
RLU: Relative light units
RNS: Reactive nitrogen species
ROS: Reactive oxygen species
SOD: Superoxide dismutase
TBST: Tris buffered saline tween
TCID50: 50% Tissue culture infective dose
TrkB: Tropomyosin-related kinase B
