# Supplementary material for: Curcumin protects rat hippocampal neurons against pseudorabies virus by regulating the BDNF/TrkB pathway
Source: Sci Rep. 2020 Dec 17;10:22204. doi: 10.1038/s41598-020-78903-0 (PMC7746732; doi:10.1038/s41598-020-78903-0)
Supplement: Supplementary file 1 — Supplementary Information. [file 41598_2020_78903_MOESM1_ESM.pdf]

Curcumin protects rat hippocampal neurons against pseudorabies virus by regulating the BDNF/TrkB pathway

Bingjie YANG[1], Guodong LUO[1], Chen ZHANG[1], Luqiu FENG[1], Xianmei LUO[1], Ling GAN*[1,2]

[1]College of Animal Science, Southwest University, Rongchang, Chongqing 402460,China

[2]Immunology research center, Medical Research Institute, Southwest University, Chongqing, 402460,China

*Corresponding author: Ling GAN. Email: gl9089@swu.edu.cn; Full telephone:+8613983416663; Fax numbers: +86-02346751732

Supplementary figure 1

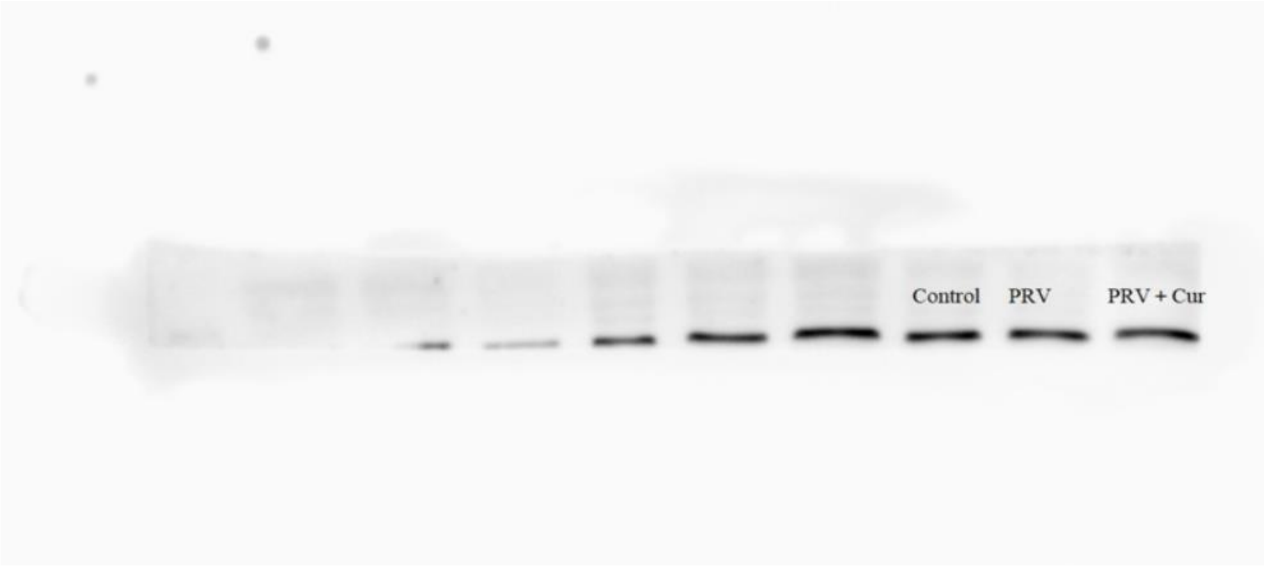

Supplementary figure 2

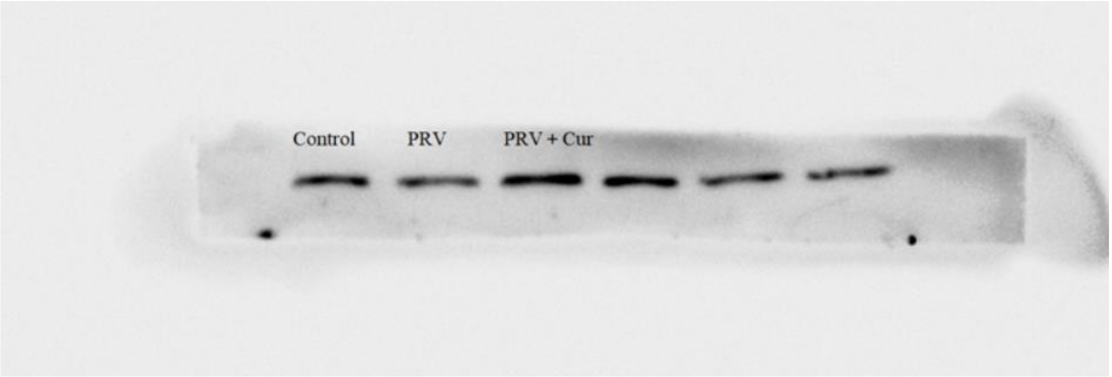

Supplementary figure 3

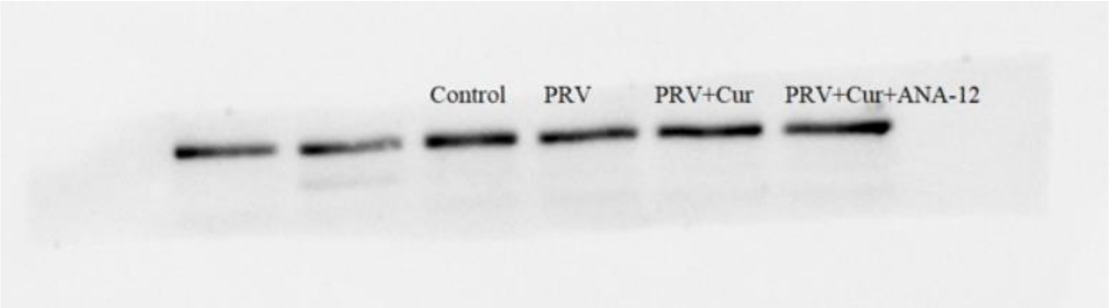

Supplementary figure 4

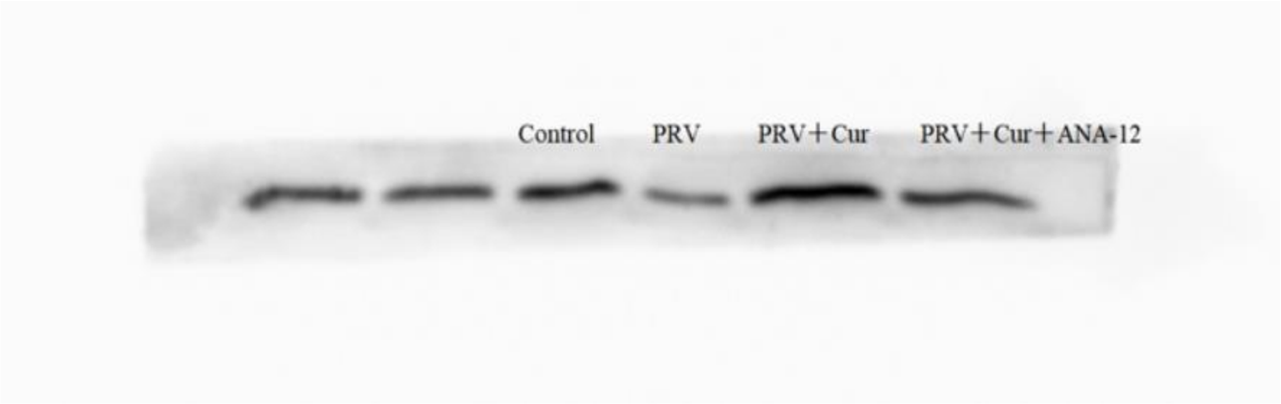

Supplementary figure 5

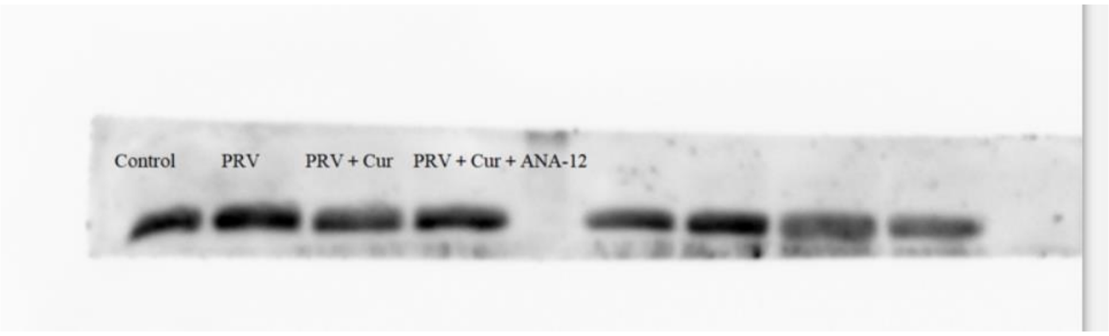

Supplementary figure 6

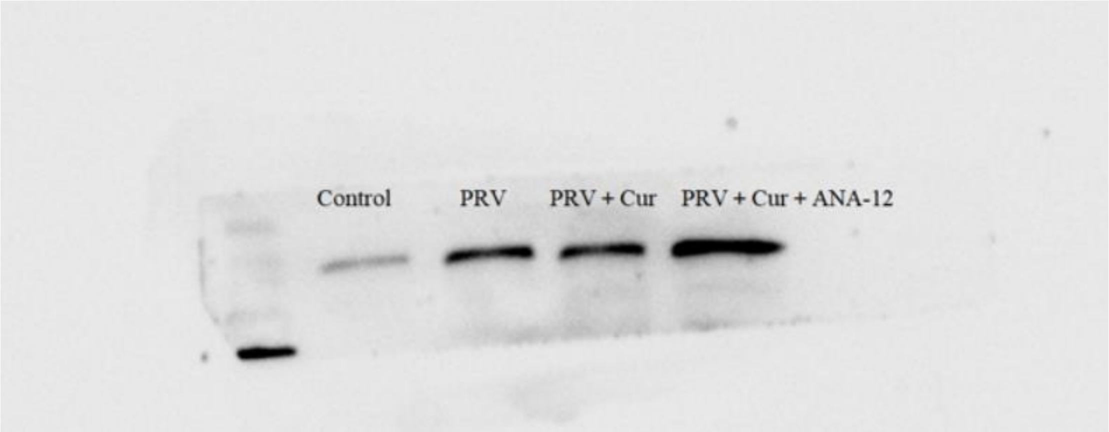

Supplementary figure 7

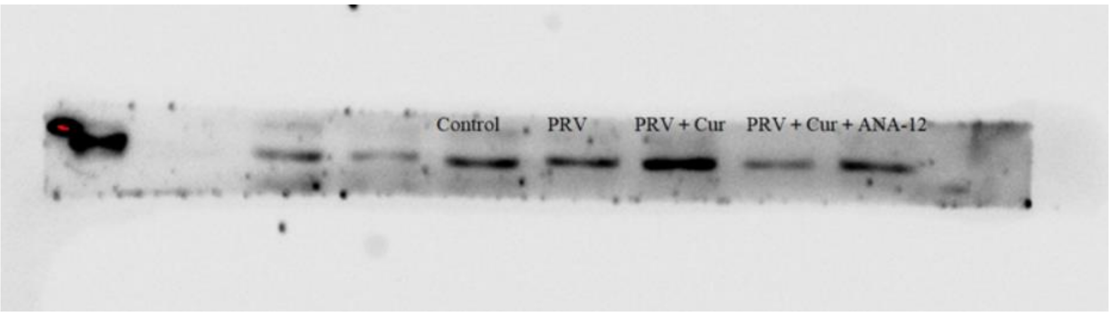

Legend

Supplementary figure 1. The original, unprocessed images of β-actin in Figure 3.

Supplementary figure 2. The original, unprocessed images of BDNF in Figure 3.

Supplementary figure 3. The original, unprocessed images of β-actin in Figure 5.

Supplementary figure 4. The original, unprocessed images of BCL-2 in Figure 5.

Supplementary figure 5. The original, unprocessed images of BAX in Figure 5.

Supplementary figure 6. The original, unprocessed images of nNOS in Figure 5.

Supplementary figure 7. The original, unprocessed images of TrkB in Figure 5.
